# Foxp3^+^CD39^+^CD73^+^ regulatory T-cells are decreased in the peripheral blood of women with deep infiltrating endometriosis

**DOI:** 10.1016/j.clinsp.2024.100390

**Published:** 2024-05-22

**Authors:** Luiza Gama Coelho Riccio, Marina Paula Andres, Isabella Zurita Dehó, Giovanna Ometto Fontanari, MaurícioSimões Abrão

**Affiliations:** a Department of Medicine, Universidade de Santo Amaro, São Paulo, SP, Brazil; b Gynecological Clinic Division, Hospital das Clínicas, Faculdade de Medicina, Universidade de São Paulo (HCFMUSP), São Paulo, SP, Brazil; c BP – Hospital Beneficência Portuguesa de São Paulo, São Paulo, SP, Brazil; d Instituto de Ensino e Pesquisa do Hospital Sírio Libanês, São Paulo, SP, Brazil

**Keywords:** Endometriosis, T Lymphocytes, Regulatory T-cells, Immunology, CD39+CD73+ suppressor Treg cells

## Abstract

Endometriosis's pathophysiology remains incompletely understood, with evidence pointing towards a dysregulated immune response. Regulatory T (Treg) cells, pivotal in maintaining self-tolerance, may facilitate the survival of ectopic endometrial cells within the abdominal cavity, thereby contributing to endometriosis development. This study aimed to assess the prevalence of CD39+CD73+ suppressor Treg cell subsets in the peripheral blood of endometriosis patients. This research focuses on the pivotal role of regulatory T-cells (Tregs), which are essential for maintaining immune tolerance and preventing autoimmune diseases. A case-control study was conducted, including 32 women diagnosed with endometriosis and 22 control subjects. The frequency of peripheral blood CD39+CD73+ suppressor Treg cells was quantified using flow cytometry. No significant differences were observed in the frequency of CD3+CD4+CD25High cells (Median [M]: 10.1; Interquartile Range [IQR]: 6.32‒18.3 vs. M: 9.72; IQR: 6.22–19.8) or CD3+CD4+CD25HighCD39+Foxp3+ cells (M: 31.1; IQR: 19.7–44.0 vs. M: 30.55; IQR: 18.5–45.5) between controls and patients. However, a significantly lower frequency of CD3+CD4+CD25HighCD39+CD73+ cells was observed in the endometriosis group compared to controls (M: 1.98; IQR: 0.0377–3.17 vs. M: 2.25; IQR: 0.50–4.08; p = 0.0483), suggesting a reduction in systemic immune tolerance among these patients. This finding highlights the potential role of CD39 and CD73 expression on Treg cells as biomarkers for assessing disease severity and progression. Furthermore, elucidating the mechanisms driving these alterations may unveil new therapeutic strategies to restore immune equilibrium and mitigate endometriosis symptoms.

## Highlights

• Tregs maintain self-tolerance and may allow the survival of ectopic endometrium.
• CD3^+^CD4^+^CD25^High^ cells were similar between endometriosis and controls.
• CD3^+^CD4^+^CD25^High^CD39^+^Foxp3^+^ cells were similar between endometriosis and controls.
• Foxp3^+^CD39^+^CD73^+^ Tregs are decreased in the blood of women with endometriosis.
• There is a decrease in systemic immune tolerance in patients with endometriosis.

## Introduction

Recent advancements in immunology have sparked a growing interest in exploring the immune system's intricate role in endometriosis, positioning studies like ours at the forefront of uncovering potential immunological dysfunctions contributing to the disease's pathogenesis. Endometriosis is one of the most common gynecological disorders, affecting 10 % to 15 % of women of reproductive age. It is a chronic, benign disease characterized by the presence of endometrial glands and stroma at extrauterine sites. Patients experience pelvic pain and 30 % to 50 % of women with the disease are infertile.^1^

The pathophysiology of endometriosis is not completely understood, and the most accepted theory – retrograde menstruation – was proposed by Sampson.^2^ Although 90 % of women have retrograde menstruation flow, only 10 % develop the disease. Recent evidence suggests that a dysregulated immune response in endometriosis is likely to originate within the eutopic endometrium.^3^ Endometriosis develops when a deficiency in the local immune response occurs, whilst its progression depends on the intensity of this process.^4^ In this context, as regulatory T-cells are involved in the maintenance of self-tolerance, they may allow the survival of endometrial cells in the abdominal cavity, and therefore be involved with the development of endometriosis.^3^

Following the discussion of immune tolerance mechanisms in endometriosis, it is imperative to delve into the specific role and implications of regulatory-T (Treg) cells within this context. Tregs, characterized by the expression of markers such as Foxp3, CD39, and CD73, serve a fundamental role in suppressing aberrant immune responses and maintaining self-tolerance. Their dysfunction or dysregulation in endometriosis could lead to the unchecked proliferation of ectopic endometrial cells, suggesting a critical breakdown in the body's ability to maintain immune homeostasis. This insight into the function and potential dysregulation of Tregs in endometriosis not only bridges the gap between complex immunological concepts and their clinical implications but also opens new avenues for targeted therapeutic interventions aimed at restoring immune balance and mitigating the disease's manifestations.^3^^,^^4^

Regulatory T-cells are specialized cells derived from a lineage of CD4 T-cells that have the crucial role of suppressing the immune response including cellular proliferation and cytokine release. Intracellular expression of Forkhead Box P3 (Foxp3) has a defining role in regulating the development and maintenance of the tolerance function of regulatory T-cells. There are many surface molecules linked to Treg cell suppression, among which CD39 and CD73 have been recently described by Deaglio et al.^5^

The combination between the surface markers CD25^+^ and CD39^+^ characterizes Foxp3+ Treg cells with immunosuppressive capacity. The mechanisms by which Treg cells can mediate immunosuppression include CD39 and CD73, ecto-enzymes, and surface markers highly expressed on Treg Foxp3+ cells.^6^

CD39 expression plays a key role in the immunosuppressive function of Treg cells.^7^ The functional molecules CD39 and CD73 of Tregs are important to the growth of ectopic lesions and with the ability of endometrial cells to invade adjacent tissues in endometriosis. CD73 is related to the increased expression of Matrix Metalloproteinase-2 (MMP2) that is related to this tissue invasion.

There are different subtypes of T-lymphocytes with immunosuppressive capacity, which differ from each other by phenotype, tissue of origin, and cytokine secretion. Among them, the authors can cite: CD4^+^CD25^+^ regulatory T-cells, which are probably the lineage with the most potent immune system suppression;^8^^,^^9^ type 1 or Tr1 regulatory T-cells (CD4^+^CD25^+^Foxp3^−^), which perform suppression through IL-10;^10^ and T Helper 3 or Th3 cells (CD4^+^CD25^−^Foxp3^+^), which induce immunity inhibition through TGF-beta.^11^

An increased expression of Foxp3^+^ cells in ectopic lesions and the presence of abundant Treg cells in the periphery of deep endometriotic lesions have been described, suggesting that these cells probably contribute to the progression of the lesions.^12^^,^^13^ Also, elevated expression of Treg cells in endometriotic lesions has already been reported.^12^^,^^14^ However, the exact mechanism by which they reach these sites and the association with clinical symptoms is still unclear.

The present study aimed to analyze the presence of CD39^+^CD73^+^ suppressor regulatory T-cells and their subsets in the peripheral blood of patients with endometriosis.

Recent studies have highlighted the multifaceted role of immune dysregulation in the pathogenesis of endometriosis. Immune dysregulation in endometriosis is characterized by altered functions of various immune cells, including macrophages, T-cells, and Natural Killer (NK) cells, leading to an environment conducive to the survival and growth of ectopic endometrial cells. Specifically, the impaired cytotoxic activity of NK cells, the skewed T-cell responses favoring Th2 over Th1 cytokine production, and the enhanced activity of regulatory T-cells (Tregs) to suppress the immune response against ectopic endometrial cells are key features. Moreover, the production of pro-inflammatory cytokines and growth factors by activated macrophages and other immune cells contributes to the proliferation and angiogenesis of endometriotic lesions, further complicating the immune landscape in endometriosis. Understanding these immune dysregulation mechanisms not only sheds light on the disease's pathophysiology but also opens avenues for novel therapeutic strategies targeting the immune system to manage endometriosis effectively.

## Materials and methods

This case-control study, conducted following the STROBE Statement, enrolled 32 women with endometriosis, confirmed by histological analysis through laparoscopy, and 22 women with no evidence of endometriosis during laparoscopy for tubal ligation. This study was approved by the Ethics Committee of Hospital das Clinicas, Faculdade de Medicina, Universidade de São Paulo (CAppesq approval number 0386/11). All women signed an informed consent form prior to participating in the study.

The sample size for this study was meticulously calculated based on a power analysis to ensure the statistical significance and reliability of the present findings. Considering a type I error rate of 5 % and aiming for a power of 80 %, the authors determined that a minimum of 63 participants per group was necessary. This calculation was predicated on prior research indicating the expected variance in Treg levels between individuals with and without endometriosis, thereby allowing us to discern a meaningful difference in Treg populations with confidence. This methodological rigor supports the validity of these conclusions regarding the immunological disparities observed in endometriosis patients.

Were included in the Endometriosis group of women between 18 and 45 years old with histological findings of deep infiltrating endometriosis of the bowel; without any autoimmune disease, confirmed by their medical history, clinical exams, and laboratory tests, when necessary; they all had regular menstrual cycles, lasting between 26 and 34 days, and none of them had any hormonal treatment in the three months prior to sample collection. Patients with active gynecological infections or irregular menstrual cycles were excluded. Healthy controls were matched in the same criteria, except they had no visualization of endometriosis in any abdominal site during laparoscopy.

In the present study, the control group comprised 22 women undergoing laparoscopy for tubal ligation, with no evidence of endometriosis or other pelvic pathologies confirmed during the procedure. The selection criteria for these controls were meticulously aligned with those of the endometriosis group to ensure comparability. Specifically, controls were matched based on age, menstrual cycle regularity, and absence of autoimmune diseases, as verified through medical history, clinical examination, and necessary laboratory tests. Exclusion criteria for the control group mirrored those of the endometriosis group, excluding individuals with active gynecological infections, irregular menstrual cycles, or any hormonal treatment within three months prior to sample collection. This stringent selection process was designed to minimize confounding factors and enhance the reliability of the comparative analysis between women with and without endometriosis.

### Obtaining peripheral blood mononuclear cells

Heparinized peripheral blood was centrifuged (Eppendorf Centrifuge model 5810R) at 3000 rpm at 10°C for 8 minutes. The plasma was discarded. The total leukocyte precipitate was removed and washed with physiological solution (previously heated in a water bath at 37°C), and centrifuged again at 3000 rpm at 10°C for 8 minutes. The supernatant was discarded. Then, the cells were placed in 2 mL of DMEM culture medium (Gibco®, ThermoFisher Scientific, Massachusetts, USA) containing 10 % fetal bovine serum (previously heated in a water bath at 37°C) for cell counting, in a Neubauer chamber. Total leukocytes were stored in liquid nitrogen in aliquots containing 2 × 106 cells in 1 mL of freezing medium (fetal bovine serum + 5 % DMSO) at room temperature (20‒26°C).

### Thawing of peripheral blood mononuclear cells

This procedure was performed in a level 2 biological safety cabinet, previously sterilized for 15 minutes under ultraviolet light. Cryopreserved blood samples were kept on dry ice until thawing. Cryotubes were partially thawed in a water bath at 37°C. 1 mL of R10 culture medium was added to the cells, dripping slowly. Afterward, the total content of the sample was transferred to a conical tube containing R10, also using the drip procedure. To carry out the washing of the cells, an additional 1 mL of R10 was added and this volume was transferred to the tube containing R10. Then, the tube was centrifuged for 10 minutes at 250 × g at a temperature of 4° to 6°C; the supernatant was discarded and 2 mL of R10 was added, to perform the cell count through the Neubauer chamber and the Countess™ automated cell counter (Invitrogen, ThermoFisher Scientific, Massachusetts, USA) using the same procedures and calculations described above. The tube was centrifuged, and the cells resuspended in R10 in the volume of 1.0 × 10^6^ cells/mL and were transferred to a 96-well V-bottom plate (NUNC® Brand Products, Sigma-Aldrich Corporation, Missouri, USA).

### Cell surface immunophenotyping in peripheral blood mononuclear cells

To characterize the CD39^+^ suppressor regulatory T-cells from peripheral blood, the collected samples were processed for cell surface immunophenotyping using the flow cytometry technique. After transferring the thawed cells to a 96-well V-bottom plate (NUNC® Brand Products, Sigma-Aldrich Corporation, Missouri, USA), 150 µL of PAB buffer was added and the plate was centrifuged at 1800 rpm for 5 minutes for cell washing. To initiate the labeling of cell surface proteins, monoclonal antibodies (CD4, CD8, CD3, CD25, CD127, CD39, CD73) were added previously titrated. The cells remained under incubation for 30 minutes in the dark at room temperature (20‒26°C). In cell surface immunophenotyping procedures for peripheral blood mononuclear cells, the authors utilized a panel of monoclonal antibodies specifically chosen for their relevance to identifying suppressor regulatory T-cell subsets, including CD39+ suppressor regulatory T-cells. The antibodies used were as follows: anti-CD4-FITC, anti-CD8-PerCP, anti-CD3-APC, anti-CD25-PE, anti-CD127-PE-Cy7, anti-CD39-APC-Cy7, and anti-CD73-BV421. Each antibody was titrated to determine the optimal dilution that provided the best signal-to-noise ratio before being used in the study. After the incubation with these antibodies, cells were washed and fixed for flow cytometric analysis. This step was critical for ensuring that the cells were adequately prepared for the detection of intracellular FoxP3 expression, which was accomplished using an anti-FoxP3-PE antibody following permeabilization of the cells. The flow cytometry analysis was performed on a BD LSRFortessa™ cell analyzer, and data were analyzed using BD FACSDiva™ software. This comprehensive approach to immunophenotyping allowed for the accurate identification and enumeration of the CD39+CD73+ regulatory T-cell populations, crucial for the present study's objective to elucidate their role in endometriosis.

Thus, cells were centrifuged and resuspended in 130 mL of FoxP3 fixative/permeabilizer and left for 30 minutes in the dark at room temperature (20‒26°C). Then, they were centrifuged and resuspended in 200 mL of 1 × permeabilization solution (the 10 × Permeabilization Buffer was diluted to 1 × , thus 60 mL of permeabilization buffer in 540 mL of deionized water) and centrifuged at 1800 rpm for 3 minutes at 22°C. After this centrifugation cells were labeled with the antibody of interest FoxP3, then incubated in the dark for 1 hour at room temperature (20‒26°C). After this period, the cells were washed twice in a diluted buffer from the ebioscience Kit (ThermoFisher Scientific, Massachusetts, USA), centrifuged for 5 minutes at 1200 rpm and cells resuspended in FACS Buffer. Samples were read using the flow cytometer.

### Immunophenotyping analysis

**Fig. 1:**
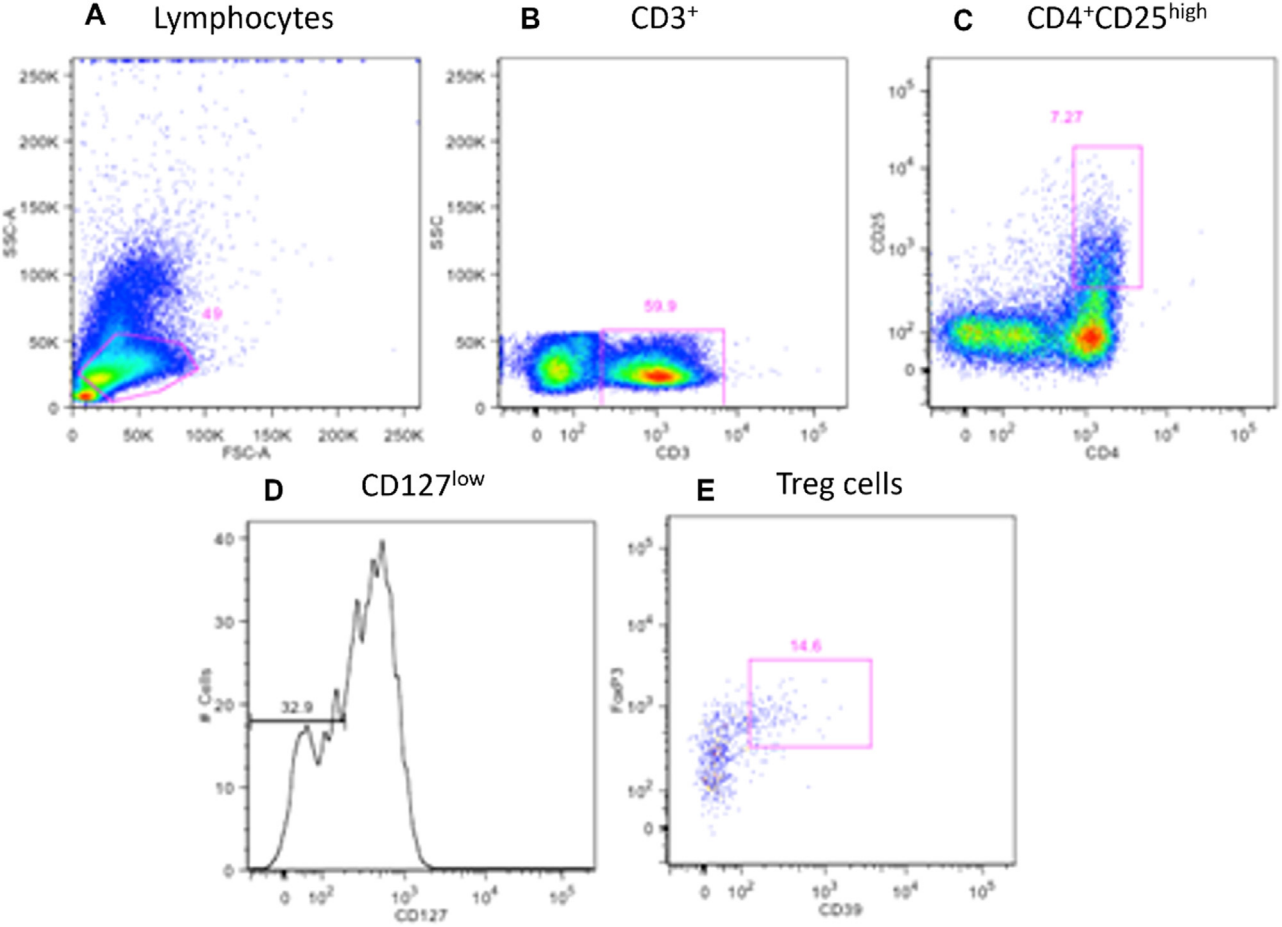
Flow cytometry gating strategy. (A) Selection of total lymphocytes (49 %). (B) CD3+ lymphocytes selection (59.9 %) from total lymphocytes. (C) From these CD3+ T-lymphocytes, double-positive populations for CD4 and CD25^high^ (7.27 %) were selected. (D) It was confirmed that 32.9 % of this population was CD127^low^. (E) Among those, cells that expressed both CD39 and FoxP3 were defined as regulatory T (Treg) suppressor cells.

**Fig. 2:**
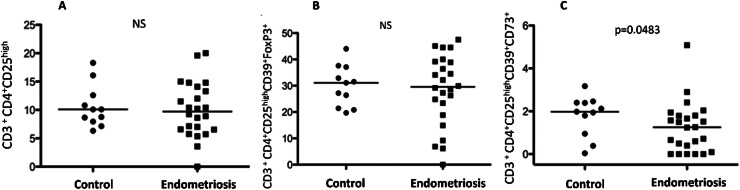
(A) Regulatory T-cells (Tregs) CD3+CD4+CD25^High^ in peripheral blood of healthy controls and women with deep infiltrating endometriosis. (B) Regulatory T-cells (Tregs) CD3+CD4+CD25^High^CD39+Foxp3+ in peripheral blood of healthy controls and women with endometriosis. (C) Regulatory T-cells (Tregs) CD3+CD4+CD25^High^CD39+CD73+ in peripheral blood of healthy controls and women with deep infiltrating endometriosis.

Representative flow cytometry analyzes monoclonal antibody labels: CD3, CD4, CD8, CD25, CD127, and CD39 (Figs. 1 and 2).

### Outcomes evaluated

Clinical pain symptoms (dysmenorrhea, deep dyspareunia, non-cyclic pelvic pain, cyclic dyschezia, and cyclic dysuria) were evaluated using a visual analog scale from 0 (no pain) to 10 (worst pain). Infertility was defined as the absence of pregnancy after one year of unprotected sexual intercourse. During surgery, endometriosis was classified according to the American Society for Reproductive Medicine ^15^ in stages I‒IV.

### Statistical analysis

Numerical data was described as mean ± standard deviation or Median (M) and Interquartile Range (IQR). Groups were compared using ANOVA with Bonferroni post-hoc test or Student's *t*-test. Categorical data was described as absolute and relative frequencies and compared with the Chi-Square test or Fisher's exact test; p-values < 0.05 were considered significant.

In the statistical analysis of these results, the authors employed various tests to accurately assess the differences between the control and endometriosis groups, taking into account the type of data and distribution. For continuous variables that followed a normal distribution, the authors used the Student's *t*-test to compare the two groups. In cases where the data were not normally distributed, comparisons were made using the Mann-Whitney *U* test. For the analysis of categorical data, the Chi-Square test or Fisher's exact test was applied depending on the expected frequencies in each category. ANOVA with Bonferroni post-hoc test was used for comparisons involving more than two groups or categories for normally distributed variables. Each test was chosen based on the best statistical practice for the type of data being analyzed, ensuring that these findings are robust and reliable; p-values less than 0.05 were considered statistically significant.

## Results

In this study, the authors evaluated 32 women diagnosed with endometriosis and 22 healthy controls. The baseline characteristics, including age and menstrual cycle phase, showed no significant differences between the groups, ensuring comparability. However, a notable difference was observed in Body Mass Index (BMI), with the endometriosis group presenting a lower mean BMI compared to controls (22.5 ± 3.2 vs. 28.9 ± 6.3 kg/m^2^; p < 0.001) (Tables 1 and 2).

**Table 1:** Characteristics of patients.

	**Endometriosis (n** = **32)**	**Control (n** = **22)**	**p**
**Age (years)**	35.3 ± 6.6	35.3 ± 5.8	>0.999^a^
**BMI (Kg/m^2^)**	22.5 ± 3.2	28.9 ± 6.3	<0.001^a^
**Race n (%)**			0.410^b^
Caucasian	26 (81.2)	7 (31.8)	
Black	3 (9.4)	0 (0)	
Mixed	1 (3.1)	1 (4.5)	
Asian	1 (3.1)	1 (4.5)	
Not reported	1 (3.1)	13 (59.0)	
**Menstrual cycle phase**			0.376^b^
Proliferative	8 (25.0)	8 (36.4)	
Secretory	21 (65.6)	10 (45.5)	
Amenorrhea	0 (0)	1 (4.5)	
Not reported	3 (9.4)	3 (13.6)	
**Pain Symptoms (VAS)**			
Dysmenorrhea			
Mean ± SD	6.7 ± 3.0	1.1 ± 2.7	<0.001^a^
n (%)	28 (87.5)	4 (18.2)	<0.001^b^
Dyspareunia			
Mean ± SD	3.2 ± 3.6	1.3 ± 3.0	0.04^a^
n (%)	16 (50.0)	4 (18.2)	0.001^b^
**Non-cyclic pelvic pain**			
Mean ± SD	2.6 ± 3.4	1.0 ± 2.5	0.06^a^
n (%)	13 (40.6)	4 (18.2)	0.08^b^
**Cyclic dysuria**			
Mean ± SD	0.2 ± 0.9	0.5 ± 2.0	0.458^a^
n (%)	1 (3.1)	2 (9.1)	0.347
**Cyclic dyschezia**			
Mean ± SD	2.3 ± 3.4	0.6 ± 2.3	0.04^a^
n (%)	11 (34.4)	2 (9.1)	0.03^b^
**Infertility n (%)**	14/20 (70.0)	0 (0)	‒
**ASRM Stage n (%)**			
I	3 (9.4)	‒	
II	6 (18.7)	‒	
III	5 (15.6)	‒	
IV	18 (56.3)	‒	

ASRM, American Society of Reproductive Medicine; BMI, Body Mass Index; n, number; VAS, Visual Analog Scale from to 0–10.

a Student's *t*-test.

b Chi-Square test.

**Table 2:** Clinical characteristics of patients in endometriosis group.

	**Endometriosis (n** = **32)**	**Control (n** = **22)**	**p**
**Symptoms (VAS)**			
Dysmenorrhea	6.7 ± 3.0	1.1 ± 2.7	<0.001
Dyspareunia	3.2 ± 3.6	1.3 ± 3.0	0.04
Non-cyclic pelvic pain	2.6 ± 3.4	1.0 ± 2.5	0.06
Cyclic dyschezia	0.2 ± 0.9	0.5 ± 2.0	0.458
Bowel symptoms	2.3 ± 3.4	0.6 ± 2.3	0.04
Infertility	14/20 (70.0)	0 (0)	‒
**Phenotype n (%)**			
Superficial	20 (6.25)	‒	
Ovarian endometrioma	6 (18.75)	‒	
Deep endometriosis	4 (12.5)	‒	
**ASRM classification n (%)**			
Stage I	3 (9.4)	‒	
Stage II	6 (18.7)	‒	
Stage III	5 (15.6)	‒	
Stage IV	18 (56.3)	‒	
**Histological classification n (%)**			
Stromal	3 (9.4)	‒	
Stromal + Mixed	8 (25.0)	‒	
Stromal + Indifferenciated	18 (56.3)	‒	
Well differenciated	1 (3.1)	‒	
Not reported	2 (6.2)		

ASRM, American Society of Reproductive Medicine; BMI, Body Mass Index; n, number; VAS, Visual Analog Scale from to 0–10.

Pain assessment revealed significantly higher scores for dysmenorrhea, deep dyspareunia, and cyclic dyschezia in patients with endometriosis compared to controls, highlighting the disease's impact on quality of life. The distribution of endometriosis stages among patients underlined the severity of the condition, with a majority in stage IV (56.3 %).

Flow cytometric analysis focused on the frequency of various Treg cell subsets in peripheral blood. No significant differences were observed between patients and controls in the frequencies of CD3+CD4+CD25High and CD3+CD4+CD25HighCD39+Foxp3+ cells. However, a significant decrease in CD3+CD4+CD25HighCD39+CD73+ cells was noted in the endometriosis group (M: 1.98; IQR: 0.0377–3.17 vs. M: 1.25; IQR: 0–5.08; p = 0.0483), suggesting a potential alteration in systemic immune tolerance mechanisms in endometriosis.

These findings contribute to understanding of the immunological landscape in endometriosis, indicating a specific decrease in a Treg cell subset that may influence the disease's pathophysiology. The results underscore the complexity of endometriosis-related immune alterations and pave the way for further research into targeted therapeutic strategies.

## Discussion

Increased expression of Foxp3+ in the ectopic endometrium was first described in 2009 by Budiu et al.^14^ Later, Braundmeier et al.^12^ reported that these cells seem to contribute to the infiltration of ectopic tissue, as increased Treg cells were observed in deep lesions, displayed in a peripheric pattern, that suggests a direct recruitment of these cells by the lesions.

The most prevalent symptom observed in the patients with endometriosis was dysmenorrhea (87.5 %). It is known that pain symptoms are very common in women with endometriosis when compared to the general population.^1^^,^^16^ In addition, most of the patients included in the study (56.3 %) presented the disease in stage IV, which is frequently associated with severe pain symptoms.

Infertility was present in 70 % of the patients with endometriosis included in the study, compared to a 30 %‒50 % rate reported in the literature.^17^ Many altered aspects seem to be related to infertility in women with endometriosis, such as Fallopian tube function, oocyte quality, folliculogenesis, spermatozoa function, endometrial receptivity, and embryo development.^18^

It was demonstrated that the variations in Treg cell population in the peripheral blood during menstrual cycle phases are important to reproductive processes,^19^ as well as Treg cells in human endometrium for maintaining immunological homeostasis within the uterus. These cells also play a key role in the maintenance of maternal-fetal tolerance during pregnancy, as the deregulation of their frequency in women with recurrent spontaneous abortion may contribute to reproductive failure.^19^

Previous studies only found an association of Foxp3 expression in the endometrium of patients with mild endometriosis in the peri-implantation period.^20^ Interestingly, patients with preeclampsia and recurrent miscarriages have decreased Treg cells in peripheral blood and decidua compared to women with normal fertility, which may be related to a lower immunosuppressive capacity and a predisposition to pregnancy loss.^9^ This fact can be related to the present findings, due to the lower frequency of CD39^+^CD73^+^ Treg cells in the peripheral blood of patients with endometriosis.

The mechanism by which Treg cells differentiate and reach endometriotic lesions is still unclear. Two models were proposed to explain the increase of leukocytes in ectopic lesions: (1) Endogenous model: the heritage of factors that promote the development of regulatory leukocytes in the eutopic endometrium allows the survival of endometrial fragments in the peritoneal cavity; and (2) Exogenous model: suggests that all women may present leukocytes in the uterine cavity, but some factors of the peritoneal microenvironment in endometriosis induce their differentiation into a regulatory phenotype. In both cases, ectopic lesions can survive, differentiate and invade the serosa.^21^

The previous report of the difference in frequency of these cells according to the different compartments of the body makes it important to study each one of them separately. Increased Treg cells in the peritoneal fluid are also related to the immunotolerance present in endometriosis and to the disease's progression.^22^ This increase in peritoneal fluid is even more evident in patients with stage III and IV endometriosis.^23^

Hanada et al.^24^ demonstrated that high levels of TGF-beta can be detected in the peritoneum of patients with endometriosis. Sbracia M, et al.^25^ reported the expression of the Pre-Implantation Factor, as well as the increased expression of FoxP3+ at the same site. These results support the hypothesis that decreased FoxP3+ expression in peripheral blood is due to the recruitment of these cells to the lesion site.

The observed decrease in Foxp3+CD39+CD73+ Treg cells in the peripheral blood of endometriosis patients may have profound implications for the pathophysiology of the disease. Regulatory T-cells play a pivotal role in maintaining immune homeostasis and tolerance by suppressing excessive immune responses that could lead to tissue damage. The reduction of these cells in the peripheral circulation suggests a compromised systemic immune tolerance in endometriosis, potentially facilitating the survival and proliferation of ectopic endometrial tissue. Furthermore, the imbalance between pro-inflammatory and anti-inflammatory forces, exemplified by the decreased presence of immunosuppressive Treg cells, could exacerbate the inflammatory environment characteristic of endometriosis. This inflammation, in turn, may contribute to the development and persistence of endometriotic lesions, pain, and infertility associated with the condition. The specific decrease in CD39+CD73+ Treg cells is particularly significant, given the role of these markers in adenosine production, a potent anti-inflammatory and immunosuppressive molecule. Thus, the diminished frequency of CD39+CD73+ Treg cells may reflect not only a decrease in systemic immune tolerance but also a reduced capacity to counteract inflammatory processes within the pelvic environment, further supporting the hypothesis that targeting these cells could offer therapeutic benefits.

Concerning the frequency of these cells in peripheral blood, the findings in the literature are inconclusive and conflicting.^3^^,^^12^ In the present study, when the authors used the CD3^+^CD4^+^CD25^high^ phenotype to isolate Treg cells from peripheral blood, we found no statistically significant difference in the frequency of these cells between controls and women with endometriosis.

Takamura et al.^26^ characterized Tregs as CD4^+^CD25^+^Foxp3^+^ cells, and also did not find statistically significant different values between the two groups. Delbandi, et al.^27^ even found high levels of Treg cells in the peripheral blood of women with endometriosis. However, it is known that the CD4^+^ and CD25^+^ markers contain populations of Tregs and effector T-cells ^6^ and Foxp3^+^ is not restricted to Tregs and may be also activated in other cell types, such as effector T-cells and tumor cells.^28^^,^^29^ Furthermore, the sample used in their study was small.

The characterization of Treg cells using CD4^+^CD39^+^Foxp3^+^ manages to isolate more than 90 % of these cells. Furthermore, CD39 and CD73 expression in Tregs can be modulated when in cultures with macrophages and endometrial tissues.^23^ However, in the present study, when the authors added the CD39 marker to characterize Treg cells, the difference in their number in the peripheral blood of patients compared to controls was not statistically significant.

Conventional T-cells have a low expression of CD39, while Treg cells are strongly positive for CD39 and CD73 expression.^7^ This is the first study to use the double labeling CD39^+^CD73^+^Foxp3^+^ to verify the frequency of Treg cells in peripheral blood. Through this marking, the authors were able to find a statistically significant difference in the frequency of Treg cells in peripheral blood, which was lower in patients with deep endometriosis. However, this study has a small sample size as a limitation, and further investigation including more patients could demonstrate statistically significant differences in other immunoregulatory cells in the peripheral blood of women with endometriosis.

To confirm the present hypotheses, it is also necessary to verify the frequency of these cells in ectopic lesions. However, so far the authors can assume that the decrease in Treg cells in peripheral blood occurs due to the migration of these cells to the lesion sites, since the increase in expression of CD39 stimulates the entry of these cells into inflamed tissues.^30^ In addition, the lesions are characterized by elevated levels of Natural Killer cells, decreased macrophage phagocytic capacity, immature dendritic cells and T-cells.^13^

As described above, studies that did not use the association of CD39 and CD73 in their Treg cell isolation strategy did not achieve statistically significant differences in the frequency of Tregs in peripheral blood between the two groups.^14^^,^^26^ The only one that also found decreased peripheral Treg cells was that of Braundmeier et al.^12^ but the study evaluated primates. However, their results are relevant, since Treg cell levels were compared in the same animal before and after disease onset. As these findings highlight a significant decrease in CD39+CD73+ regulatory T-cells in the peripheral blood of women with endometriosis, they open up new avenues for therapeutic intervention targeting these cells. Given the crucial role of CD39 and CD73 in mediating the immunosuppressive function of Treg cells, enhancing their activity or frequency could offer a novel strategy to restore immune tolerance and potentially mitigate the progression of endometriosis. This perspective warrants further investigation into targeted therapies that could modulate Treg cell function as a means to address the underlying immunological dysfunctions in endometriosis.

Interestingly Jiang, et al.^31^ demonstrated that with the reduction of myeloid suppressor cells, through the inhibition of the Notch pathway, the progression of endometriosis can be avoided. This can be a potential target of studies for new therapeutic strategies for the disease.

The observed reduction in CD39+CD73+ Treg cells in the peripheral blood of patients with endometriosis offers a novel perspective on the disease's immunological dysfunction and presents an intriguing avenue for therapeutic intervention. Given the critical role these cells play in maintaining immune homeostasis, their diminution suggests a targeted disruption of immune tolerance mechanisms in endometriosis, which might contribute to the disease's persistence and symptomatology. Targeting pathways to enhance or restore the function and number of these Treg cells could potentially rebalance the immune system, reduce inflammation, and thereby alleviate the clinical manifestations of endometriosis. For instance, strategies aimed at increasing adenosine production, a process in which CD39 and CD73 are key enzymes, could mitigate the inflammatory milieu associated with endometriosis. Such approaches could include the use of pharmacological agents that upregulate CD39 and CD73 expression or activity, or cell therapy techniques to enrich Treg populations with high CD39 and CD73 expression in the peripheral blood. Moreover, understanding the migration patterns and functional changes of CD39+CD73+ Treg cells in endometriosis might reveal additional targets for modulating the immune response in this condition. Further research in this area could pave the way for innovative, immunologically based treatments that offer relief to patients suffering from this debilitating disease.

## Conclusions

This study has demonstrated a notable decrease in the levels of Treg Foxp3+CD3+CD4+CD25HighCD39+CD73+ cells in the peripheral blood of women with endometriosis compared to healthy controls. This finding points to a potential decrease in systemic immune tolerance in patients with endometriosis, given the high expression of CD39 and CD73 in Treg cells with immunosuppressive functions. The specificity of this decrease suggests a unique immunological fingerprint of endometriosis, underscoring the complex interplay between immune regulation and the pathophysiology of the disease.

Future research should expand upon these findings by exploring the frequency and function of these cells in ectopic lesions and the endometrium to confirm their immunosuppressive action in the progression of endometriosis. Given the pivotal role of Treg cells in mediating immune tolerance, a deeper investigation into how these cells influence various forms of the disease, including peritoneal, ovarian, and deep infiltrating endometriosis, is warranted. Moreover, elucidating the therapeutic potential of modulating Treg cell function offers a promising avenue for the development of novel treatments for endometriosis. Strategies could include targeting the CD39 and CD73 pathways to enhance the immunosuppressive capacity of Treg cells, potentially mitigating the inflammatory response and improving symptomatology in endometriosis patients. Such research could yield significant insights into the immunological underpinnings of endometriosis and foster the development of innovative therapeutic modalities tailored to modulate the immune environment.

## Declaration of competing interest

The authors declare no conflicts of interest.
